# Testing for Individual Differences in the Identification of Chemosignals for Fear and Happy: Phenotypic Super-Detectors, Detectors and Non-Detectors

**DOI:** 10.1371/journal.pone.0154495

**Published:** 2016-05-05

**Authors:** Jeannette M. Haviland-Jones, Terry R. McGuire, Patricia Wilson

**Affiliations:** 1 Psychology Department, Rutgers-The State University of New Jersey, New Brunswick, New Jersey, United States of America; 2 Genetics Department, Rutgers-The State University of New Jersey, New Brunswick, New Jersey, United States of America; 3 Psychology Department, La Salle University, Philadelphia, PA, United States of America; Technion - Israel Institute of Technology, ISRAEL

## Abstract

Mood odor identification, explicit awareness of mood odor, may be an important emotion skill and part of a complex dual processing system. It has already been shown that mood odors have significant implicit effects, effects that occur without awareness. This study applies methods for examining human individual differences in the identification of chemosignals for fear and happy, important in itself, and a key to understanding the dual processing of emotion in the olfactory system. Axillary mood odors had been collected from 14 male donors during a mood induction task. Pads were collected after 12 and 24 minutes, creating two doses. Sixty -one participants (41 females) identified the mood odor chemosignals. On a single trial, participants identified 2 doses of fear, 2 doses of happy, and a sterile control. There were 15 trials. The first analysis (r_tt_) showed that the population was phenotypically heterogeneous, not homogeneous, in identification accuracy. It also showed that a minimum of 10 trials was needed for test reliability. The second analysis, Growth Mixture Modeling, found three distinct groups of detectors: (1) 49.49% were consistently accurate super detectors, (2) 32.52% were accurate above chance level detectors, and (3) 17.98% were non-detectors. Bayesian Posterior Analyses showed reliability of groups at or above 98%. No differences related to mood odor valence (fear or happy), dose (collection at 12 or 24 minutes) or gender were found. Implications for further study of genetic differences, learning and function of identification are noted. It appears that many people can be reliable in explicitly identifying fear and happy mood odors but this skill is not homogeneous.

## Introduction

*Helen Keller wrote “… the odor of young men….suggests all the things strong and beautiful and joyous and gives me a sense of physical happiness.”*[1]

Keller may well have had “*a sense of physical happiness”* when sniffing the odor of a joyous young man, as mood odors are contagious [2, 3]. The notable aspect of this contagion is not that it happened, as there are substantial automatic or implicit effects of mood odors, but that she knew about the mood in an odor. Would her expertise have lent her more social awareness, as those who can identify the odors of close friends may have [4] or did it interact dynamically with other emotional processes related to implicit chemosensory communication (for review see [5])? Even if she were able to smell mood odor, was she a rare variation, her skill influenced by her blindness and deafness? This study is focused on the objective measurement of individual differences in identifying fear and happy mood odors. It applies accepted measures from behavioral genetics and we predict that there will be reliable individual differences in identifying mood odor.

Given that most people are dubious about the claims to sniff emotion, is it in fact rare? Or is it just not reliable—occurring at a level only slightly above chance? We [6] have shown that groups of people are slightly better than chance in identifying mood odors but are there individual differences with some people well above chance while others do not detect anything? Individual differences are ubiquitous in the human olfactory system. There are at least 400 active genes for olfactory receptors and each gene has multiple alleles. Humans may be able to discriminate more than a trillion odors [7].”In humans, genetic diversity will result in perceptual diversity. Each individual perceives olfactory stimuli with their personal set of OR s (olfactory receptors)” [8]. In other words, different people may well perceive different odors. In addition, the olfactory system has high plasticity, showing neurogenesis in the olfactory bulb [9] and in the peripheral neurons [10]. In this study we adapt accepted methods of analysis for behavioral genetics to classify phenotypically distinct groups. We examine the individual differences in identifying mood from human body semiochemicals taking some account of gender, potential dose of chemosignal, and the mood communicated.

Research on mood odors is providing a new interdisciplinary field, including sensory sciences, neurosciences, behavior genetics, and the psychology of emotion. Several recent findings illustrate how the implicit identification of mood odors is important and how individual differences in other social-emotional skills may apply. For example, De Groot and his colleagues have shown that sniffing body odor from a happy person [2] or from a fearful person [3] has an implicit effect on the mood of the person who is sniffing. It is possible that a person who also can explicitly identify the odor might respond differently from one who cannot. Zhou and Chen [4] showed that “superior skill in identifying social chemosensory information is related to higher emotional competency.” While the chemosensory information here was the odor of a person, not mood odor per se, a similar pattern may exist for mood odors with “superior skill” leading to “emotional complexity “once again. Lubke et al [11] have shown how individual differences in social openness are related to implicit effects on brain responses to mood odor. Implicitly, responses to mood odor were related to at least one kind of behavior. The identification of mood odor may be an emotion skill, not unlike the identification of mood in facial expression and differences in identification may be related to automatic behaviors as well as socio-emotional expertise (see discussion of dual process in emotion regulation [12].

When asked to identify moods from body semiochemicals [6] people seem to perform slightly better than chance. This had suggested only a minor role for the olfactory system compared to the visual or auditory systems where the moods communicated by smiles or chuckles or frowns and cries are easily identified. However, this assumption rests on tests of population means, not on tests of individuals. It also rests on one or two trials for each individual which is an acceptable approach for classifying purer and easily measured odor compounds. Neither of these methods has provided reliable information about individuals.

It is possible that there is a subpopulation of super detectors who can identify mood odors reliably, to whom the moods in body chemosignals are obvious. Equally important, however, are people who discriminate mood odors easily but who do not label or identify them correctly, a common problem for odors in general (e.g., [13]). And finally there may be people who are unable to identify the chemosignals at all. The alternative hypothesis is that there are no distinct groups, only a normal distribution of people with some range in identification skills.

### Testing for phenotypic differences

We will briefly describe two methods for testing the hypothesis that phenotypic groups exist in a population. These procedures are often used prior to the investigation of genetic differences. The first (r_tt_) tests for heterogeneity and indicates the number of trials to establish reliability [14]. The second (Growth Modeling) provides reliable models for the number of groups needed to describe a heterogeneous population [15].

#### Testing for homogeneity

It is reasonable to hypothesize that there are individual differences in olfaction. There is a large family of genes that encodes odor receptors. This family of genes [16] is the largest known among mammals in general [17]. Even for humans this is the largest gene family among the sensory systems. Variability in genomic expression is illustrated by Keller, Zhuang, Chi, Vosshall, and Matsunami [18], who have shown a connection between ability to reliably detect the chemosignals of androstenone and androstadienone (AND) and a specific genetic polymorphism. The original study exemplified a “bottom-up” approach. Using molecular techniques, the researchers found genetic variation in an olfactory receptor gene. Genotypic variation correlated with variability in the perception of androstenone. A later study in a different population [19] was not able to replicate the effects of the same genotypes. Instead, a different allele affected androstenone perception. It is quite likely that there are many patterns or pathways to the detection of the chemosignals. Nevertheless, this seminal work suggested that there are multiple genetic influences involved in the identification of human chemosensory stimuli and that people differ.

It is possible, therefore, that the identification of mood chemosignals is not homogeneous but that there are individual differences. Furthermore, individuals may fall into distinct groups that include both anosmics and super-detectors. A first step in showing further connections with mood chemosignals and genetic differences is to provide a method and evidence to examine connections to a wide variety of emerging questions. These range from the relationships between explicit and implicit reactions to mood odors, to changes in olfactory receptors related to learning, as well as to the identification of genetic subpopulations.

#### Homogeneous vs. heterogeneous testing

One classic approach is a standard behavioral genetics technique using the r_tt_ statistic [14]. Its purpose is to determine whether a population is phenotypically homogeneous or heterogeneous for a particular behavioral trait. The test is reliable even if the stimulus is not standardized, as is the case with mood semiochemicals. (Other tests could be used for standard odor compounds.) In this approach, the participants repeatedly identify the odors in a series of trials. The series provides sequences of correct or incorrect responses for each individual. The question is whether there are different types of response sequences with some individuals reaching asymptote (i.e., super-detectors) fairly quickly, for example, and other individuals (i.e., non-detectors) never making correct identifications or whether there are other patterns. Hirsch and Tryon [14] established the use of the r_tt_ statistic to describe genetic individual differences associated with behaviors that show the kind of population distribution that is seen for human mood odors (see [14], for a full discussion). If, as we predict, the r_tt_ is significant, we will have shown that there are significant individual patterns in the population worthy of further testing.

#### Number of trials

Most researchers working on mood odors, including us, have adopted an approach using just one or two trials to determine whether an individual can identify mood chemosignals. Obviously, too few trials would result in statistically unreliable results. There are reasons to avoid too many trials, however. Exposure to fear and happy mood odors [2, 3] may elicit behavior compatible with feeling fear or happiness. This suggests that moods may be affected by the chemosignals and the change in moods would possibly affect motivation and attribution decisions. It is desirable, therefore, to use as few trials as necessary to achieve reliable results. The r_tt_ statistic can suggest a minimum number of trials needed for reliable classification of individuals.

#### Modeling to establish phenotypic groups

When there is heterogeneity, a second step can be taken using longitudinal modeling to determine the number of groups and their characteristics. Such analyses are new to olfactory research although there exist several types and they are used extensively in other fields (e.g., [20, 21]). The goal is to establish the most reliable model for predicting individual belongingness to the groups [15].

In longitudinal modeling, the data from all individuals is transformed into a small number of estimated trajectory curves which correspond to groups of individuals. By Bayesian posterior probabilities, each individual’s longitudinal or series data can be assigned to a particular trajectory group. This is interpreted as assignments to specific phenotypes. The growth curve analysis allows one to detect the number of phenotypes and to calculate the reliability of assigning individuals to each phenotype. High reliability further supports the existence of distinct groups.

### The communicated mood, gender and dose of chemosignal

In this study of mood odors, the classification of individuals using behavioral genetics approaches may show that there are groups within the population who differ in their ability (a) to identify a particular mood chemosignal, i.e., fear or happy or (b) to identify a chemosignal collected after a longer exposure time from the donor rather than a shorter time. There may be (c) gender differences to consider as well. These are important secondary considerations and there is an exploration of each issue in this study

In this study we will test mood chemosignals collected from donors who have been in two mood induction conditions—fear and happy. A growing literature indicates that humans can detect fear chemosensory signals [4, 22, 23]. Fear semiochemicals augment the startle response [24], enhance cognitive performance [25], activate the amydala [26], effect the perception of ambiguous facial expressions [26, 27, 28] and affect facial responses for fear [3].

Similarly, humans may identify and respond to happiness chemosensory signals [2, 6, 22]. Less is known about related behavioral or neurological effects but both non-human pleasant odors (e.g., [29]) and human odors have effects on nonverbal behaviors. (For review, see [30, 31].)

The body of research and evolutionary implications for fear mood chemosignals versus happy ones suggests that fear signals may be more readily identified than happy emotion signals. Our study does not directly test this hypothesis as the mood chemosignals are not tested separately; however, post-hoc analyses will be used to indicate the direction of possible differences.

It is important to have sufficient chemosignal information for it to be identified. There are no standards for this presently. The excellent tests for establishing threshold levels in odor or taste detection (e.g., the staircase method, triangular bag method) usually require known quantities of pure chemicals to produce a series of measured concentrations. We address this by collecting the samples after a short period (12 minutes) and a long period (24 minutes). The longer exposure to the mood induction for the donors might increase the signal value of the chemosignal product on the axillary pads. If this is correct, the 24-minute pad has “more” semiochemical information and the detectors will have a higher rate of success in identifying it. This may be incorrect because the mood of the donor is more variable over time and thus “longer” exposure might give a more ambiguous signal, rather than a clearer signal.

The process may be even more complex as a recent study by De Groot, Smeets & Semin[32] showed. Receivers’ implicit responses were stronger to the rapidly produced chemosignal than to a chemosignal collected later. Their explanation is that these are distinct chemosignals. Until more is known about the composition of the chemosensory signals, it is wise to consider not only that the time course of the fear mood odor may vary but also that the time courses of different moods may also vary. The stronger response for fear mood odors may be rapid and stronger response for happy or other moods such as anger may be slower. In our case, the higher dose contains both the rapid and slow signals, as defined by De Groot et al. [32]. This may still provide a signal with greater explicit information for the identification of a fear signal and may still function as a type of “threshold” test.

The evidence for gender differences in decoding emotion cues tends to be relatively consistent across ages and cultures [33] and may be adaptive [34]. Women surpass men in this capacity with studies generally showing an effect size of about .20. This greater sensitivity to emotion cues may extend to mood odors. There also is evidence that women are more accurate detectors of odors in some instances [35] and particularly in the cognitive evaluation of irritating odors, though there is little evidence overall for differences in threshold sensitivity [36]. Therefore, comparisons between men’s and women’s accuracy in mood odor identification will be made. The prediction is that women will be more accurate, if there are any differences.

The variability in human genomic profiles supports an argument in favor of individual differences in human mood odor identification. We predict heterogeneity such that a small subgroup of individuals will be highly accurate though others may not detect differences. Further, the evolutionary implications for fear mood chemosignals suggests that fear signals may be more readily identified than happy emotion signals. Therefore, we predict that fear mood chemosignals are more reliably detected. Additionally, the amount of time that a donor (of axillary chemosignals) is exposed to a mood stimulus could be expected to have significant impact on the chemosignal. We predict that mood odors collected for a longer period of time will be more likely to be identified. Finally, given the body of research suggesting that women have an emotional recognition advantage, we predict that women would be more likely to be in the subgroup of detectors than men.

## Materials and Methods

### Sample preparation and participant selection

#### Chemosignal collection

In this study the mood odors were collected from 14 healthy male undergraduate nonsmokers. For a 7-day period prior to the sample collection, the donors only used the provided odor-free deodorant and cleansing products. The donors were instructed to shower (using the soap provided) the morning of sample collection approximately 6 hours prior to sample collection. They were also given a list of prohibited “spicy” and other odorous foods and did not eat them during the 24 hours prior to the collection.

Axillary samples were collected during two video mood inductions, one day apart. The fear mood and happy mood induction videos were 12-minute standardized videos (see [6, 22]). Video mood induction is highly reliable (for review see [37]). The videos were shown twice to the subjects for a 24-minute induction. The videos have multiple facial displays for fear (or happy). There is no narrative theme. This reduces the likelihood that repetition of the 12 minute video would decrease the impact of the video.

Samples were collected onto cleaned Kerlix8 brand sterile gauze. Prior to mood induction, donors were given 2 pairs of gauze strips (each strip 3cm x 8cm) in separate plastic enclosed bags labeled “right” and “left” arm. They placed one pair in each left/right axilla. At 12 minutes into the mood induction, the film was paused and donors removed one pair (1 left and 1 right) of axillary pads. Donors placed each pad into its labeled plastic zipper bags. All air was forced from the bag prior to sealing. The second pair of pads was collected in the same manner after 24 minutes. All samples were placed in a minus 80C° freezer within 2 hours of collection.

#### Preparation of odor samples

To minimize effects not related to mood condition, we combined mood odors from seven donors to make a test sample. Pads from the same donors were combined to make matching samples for fear-12, fear-24, happy-12, and happy-24. This minimizes the possibility that one donor dominates or has an “aberrant” chemosignal. A set of seven cleaned pads was used for the control. (See S1 Text for more detail.)

#### Odor detectors

Sixty-one university students and staff (41 females and 20 males) ranging in age from 18 to 28 (M age = 21, SD = 1.85) served as detectors. Detectors were nonsmokers and did not use any fragranced product on the day of the study. We did not test for general anosmia. Anosmics are improbable among a population of young adults who volunteer for an odor study. Previous testing had never revealed an anosmic detector, as expected, since smell identification performance peaks in early adulthood [38].

The entire study was approved by the Institutional Review Board of Rutgers–The State University of New Jersey. All donors and participants signed written consent forms.

### Procedure

#### Test trial

Participants (detectors) were tested individually in dedicated testing rooms approximately 8’x8’. The detector and the experimenter sat across from each other with a table between them. On each trial the experimenter placed five identical sample jars from one set of (7) donors on a plastic tray on the table, shuffled them, and presented the tray of jars to the detector. S/he was instructed to sniff the jars as many times as necessary and in any order. The detector identified the odors by setting each jar on its label (fear 12, fear 24, happy 12, happy 24, control) on a place-mat. To avoid position effects, half of the detectors had fear labels on the left side of the place mat and half of the detectors had them on the right side of the place mat. Participants were not given feedback on test trials about correct or incorrect identifications, i.e., there are no explicit training trials. A second set then would be brought forth, etc.

#### Exposure to label task

After each test trial (except the final one) the experimenter presented a set of 2 fear (or happy) samples. The experimenter explained, “These are fear (or happy); which is 12 and which is 24?” The detector made the judgment and was told if s/he was correct. This was repeated with the other mood sample. Although the task of labeling was interjected between each test trial, the result of the labeling task–labeling the dose of the samples–is analyzed separately from the identification of mood data. The purpose of the labeling task question is to have the participants attend to the mood odors in a consistent manner, always doing the same task, while being exposed to labeled odors. In no case were the fear and happy mood odors presented together for comparison and the order of presentation of the fear or happy was counterbalanced. It was important to provide the labeling task so that participants who could discriminate the samples would have a correct label (e.g., [39]). If some participants can discriminate the samples but do not correctly identify them initially, then simple exposure with labels may correct the problem.

#### Block

There were five trials testing the mood odor in a block. Between blocks participants took a 5-min break. They walked into the enclosed corridors around the testing rooms and were allowed a drink of water. There were three blocks of five trials for a total of 15 trials.

### Statistical approaches

#### Scoring

In the main analysis each of the 15 test trials was scored as correct or incorrect. To be correct both fear stimuli (collected after 12 and 24 minutes from the same donors) had to be identified as fear, both happy stimuli (collected after 12 and 24 minutes from the same donors) had to be identified as happy, and the control stimulus had to be identified as the control. Any other response set was scored as incorrect. The 12 and 24-minute samples only had to be correct for mood identification, not for dose. (The labeling task that asks only for 12 and 24 minute discrimination is examined separately in the post-hoc analyses–see below.)

#### Conservative scoring

Requiring multiple correct choices within a single trial has a lower probability of a false positive than either a single yes/no trial or a single triangular-choice trial [40]. The detectors correctly identified two fear odors, two happy odors and a control on each trial. This results in one scored “correct” trial. This provides a very low probability of false positives. Although the participants also identified which pads were collected after 12 and 24 minutes, the trial was scored correct as long as they identified the moods.

#### Description

First, we provide descriptive data to indicate the proportion of the tested population who were accurate on the first trial without exposure trials and who continued to be correct on most subsequent trials. We predict that there is a small subset of the population who can do this.

#### r_tt_

Second, we use the Hirsch-Tryon analysis method to show whether there is population heterogeneity or homogeneity [14, 41, 42]. We predict heterogeneity. If there is heterogeneity, this analysis will also indicate how many trials were necessary to establish reliable individual patterns with these mood odor stimuli. (See S2 Text for formula and further description.)

#### Growth mixture model (GMM)

Third, as one purpose of the study is to establish unique groups, we used a GMM approach. This is a statistical method that provides several key pieces of information: (1) An estimated number (*K*) of homogeneous sub-groups; (2) For each sub-group, a polynomial of order at most three (with a random error term) that describes the estimated outcome measure at a fixed set of time points and estimates the random error; (3) Bayesian posterior probabilities (BPPs) for each individual that indicate the probability that the individual belongs to each of the *K* sub-groups; (4) An estimate of the proportion of individuals in the study that belong to each of the *K* subgroups. Also, the GMM method provides a value called the Bayesian Information Criterion (BIC) that enables us to establish the best estimate of *K*, the number of homogeneous sub-groups, which is not known *a priori*. Using the BIC, we can test the null hypothesis: H_0_: *K* = 1 (Data come from one group, i.e., there is homogeneity) vs. H_a_: *K* > 1 (Data come from multiple groups, i.e., there is heterogeneity). Finally, for any specified sub-group of interest, we may use the BPPs corresponding to that subgroup as phenotypes in association studies. (In text we provide a fictitious example which clarifies how we use GMM.)

#### Post-hoc analyses

Fourth, we provide post hoc analyses. Both factor analyses and non-parametric, related samples analyses are used to describe further differences in each group. We use multivariate analysis of variance (MANOVA) to test the prediction that women have higher scores. We use the non-parametric, related samples analyses to test the prediction that all groups have higher scores on the 24 minute exposure samples, the higher dose. We use the same method to test the prediction that all groups will have higher scores on the fear samples than on the happy samples. The statistical program SPSS is used for these post hoc analyses.

## Results

### Descriptive analysis

We predicted that a small number of participants would identify mood odors consistently without any exposure/labeling experience. Eight participants (out of 61) appeared to be in this group since they were correct on the first test trial. Only two of the eight were subsequently correct on 14/15 test trials (highest score was 14/15). There were, therefore, 2/61 (3.2%) participants who correctly and consistently identified the mood odors without any exposure to labeling. Another four (6.42%) of the eight were mostly correct, having 9 to 11 of the next 14 trials correct. This still describes a very high level of accuracy and probable ability to identify mood odors reliably without exposure trials. This percentage of about 10% describes a small portion of the sample, as expected. Two were inconsistent on subsequent trials even though they were correct on the first trial.

It is clear that there are some people who can identify mood odors and who do so consistently. It is also clear that some people may be mis-classified if only one or two test trials are used. In the next section we analyze all the participants.

### *r*_*tt*_ statistic

#### Heterogeneity

The *r*_*tt*_ analysis (with 15 trials, *r*_*tt*_ = .90) supports the hypothesis that the population sampled is not homogeneous but tends towards heterogeneity (see Table 1 below). It suggests that people do have reliable patterns and they differ from each other.

**Table 1:** r_tt_ Statistic.

Trials	r_tt_
1	
2	0.50
3	0.53
4	0.69
5	0.73
6	0.75
7	0.80
8	0.82
9	0.84
10	0.85
11	0.86
12	0.87
13	0.88
14	0.89
15	0.90

#### Number of trials

The r_tt_ statistic predicts the probability of determining heterogeneity in the tested population. As can be seen in Table 1, two trials (*r*_*tt*_ = .50) clearly do not provide sufficient evidence for the reliable identification of detectors vs. non-detectors using our paradigm.

Even after 5 trials it is only .73. The trajectory is near asymptote by trial 10. At least 10 trials are needed to establish heterogeneity in identification for these mood chemosignals.

#### GMM

Growth mixture models were used to fit the series of 15 test trials to estimated trajectory curves. We conducted the growth mixture model statistics using the Bayesian Information Criterion (BIC) to determine empirically the number of trajectory groups. Since the data are binary we used a logistic model. The BIC score for *K* = 3 sub-groups was optimal; therefore, the three group solution is chosen as the best fitted model. (The BIC for a 1-group solution is BIC = -607.15; for a 2-group BIC = -485.57; for a 3-group BIC = - 480.88; for a 4-group BIC = -481.09. The 5-group failed critical assumptions, meaning one of the five polynomials did not have a significant value and had high standard error.)

**Fig 1:**
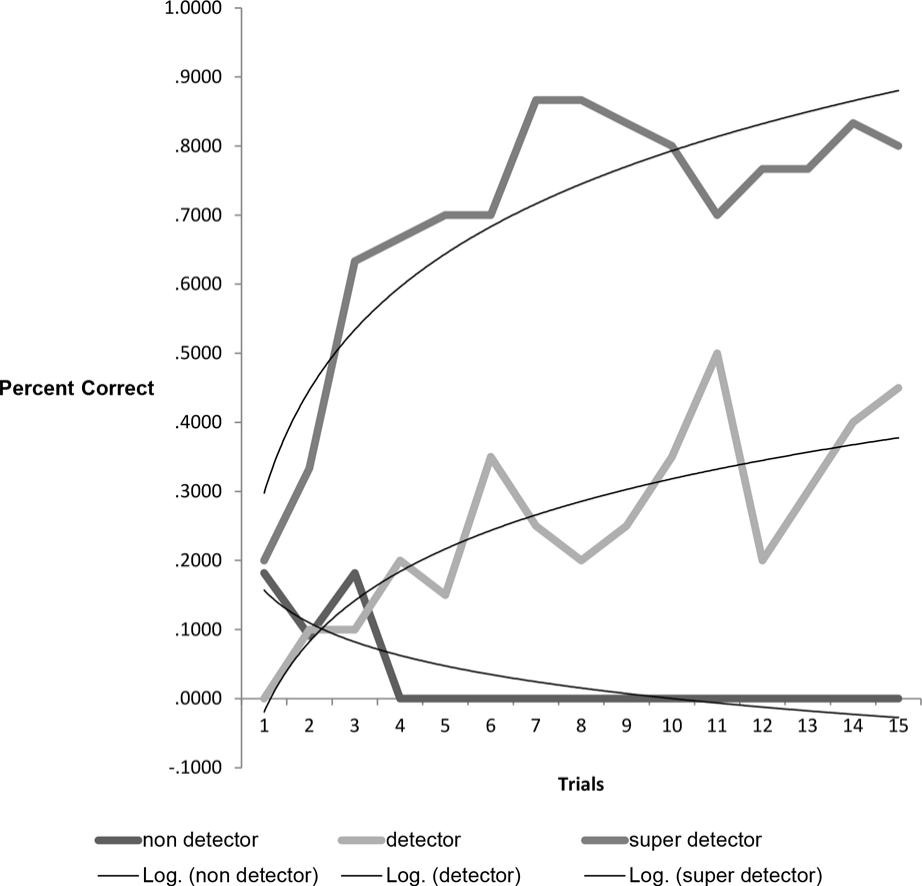
Estimated GMM Trajectories for a 3-group Solution. Fig 1 shows how individuals in each group conform to a particular trajectory. The super- detectors reach asymptote by about trial 10, becoming near perfect; detectors perform above chance; non- detectors seldom have correct trials.

The use of GMM indicates that the participants can be divided into three distinct groups. The trajectory groups were named super- detectors (49.49%), detectors (32.52%) and non-detectors (17.98%).). As shown in Fig 1, the super-detector group shows a rapid rise in the number of correct trials within the group, becoming essentially perfect in the last five trials. The non-detector group shows no change in the number of correct trials, almost never having correct trials. The detector group has correct trials at above chance levels but little change over trials.

The probability of correct assignment of individuals using the 3-group model is very high. All the non-detectors belonged to their group with a BPP of at least 0.98. Only two super-detectors fell below the .99 probability of assignment to their group; one person had .92 probability and one person had .86 probability. In both instances the alternate group assignment was to the detector group. Only two detectors fell below the .99 probability of assignment to their group; one person had .89 probability and one person had .71 probability. In both instances the alternate group assignment was to the super-detector group.

The four group solution was almost as likely to be a good model as the three group solution. In the four group model, five participants from the super-detector group were reassigned to a 4^th^ group. They differed from others in the super detector group in that they were less accurate in the last block of 5 trials rather than being more accurate. This suggests adaptation or mood interference after 10 trials. Examining the probability of belonging to a particular group in the 4^th^ group model does not decrease the number of individuals who are assigned to a group at .99 or better probability.

Over all, the size of the super- detector group is larger than predicted. Nearly half the tested population was able to discriminate and correctly identify mood odors consistently after exposure to labeled samples. There is also an unusually high level of probable assignment to the groups, further supporting the hypothesis that the groups are phenotypically distinct.

#### Model confirmation and gender analysis

The model indicates the population has three groups. A repeated measures ANOVA with the 3 groups (super-detector, detector, and non-detector) and gender as independent variables and the 3 test blocks of 5 trials each (early, middle, late) as the repeated measure shows that there is a main effect for group, as expected (*F*(2) = 154.22, *p* < .000, eta = .849). Using LSD post-hocs, each group is different from the other (p < .000). Contrary to prediction, there is no main effect for gender (*F*(1) = .099, ns). There is a significant interaction between group and the test block indicating that the trajectories of the changes are different, as expected (*F*(4) = 5.213, *p* < .001, eta = .159); see Fig 1. This analysis confirms the model of phenotypic groups but shows only a trend for gender differences

### Post-hoc analyses of mood and dose

#### Chemosignal mood

In order to test the hypothesis that the participants would identify the fear chemosignal more often than the happy chemosignal, we tested each group (super-detector, detector, and non-detector) separately comparing the errors for each mood odor that resulted from incorrectly identifying it as control. We reasoned that if one of the mood odors was more difficult to detect and identify, it would be confused with the non-odorous control. Since the super-detector group makes few errors, the chance of finding any difference in the ease of identifying one mood chemosignal over another is diminished. The detector and non-detector groups have the largest number of incorrect trials so analyzing these separately provides a better opportunity to detect trends or differences. Using the related samples Wilcoxen signed rank test, there were no significant differences between the identification of fear and happy (vs. control) in any of the groups (*p* > .268 in all cases). If the group could identify fear (vs. control), it could equally well identify happy (vs. control). Therefore, we reject the hypothesis that the fear chemosignal is more likely to be identified in this mode of testing.

In addition, we analyzed the possibility that some participants would not be able to identify the control, that is, that they could not identify any human axillary chemosignal in comparison to a sterile pad. Using the same test, there were no differences for the non-detector group in identifying the control and either mood odor (*p* > .373). The super-detectors are more likely to identify the control compared with either mood odor (*p* < .000), as are the detectors (*p* < .001). While the control (sterile pad) generally is more easily identified than the mood chemosignals, the non-detectors are not reliable in identifying it. The non-detectors not only cannot discriminate and identify the chemosignals, they also cannot identify the control.

#### Dose

Axillary body odors were collected from the donors after 12 and after 24 minutes. The hypothesis was that chemosignals collected for a longer time would be easier to identify. All tests used the Wilcoxen test for related samples. As above, each detector group was tested separately. For the fear chemosignals, there was a difference for the detector group (*p* < .048) but not for the non-detector (*p* < .715) nor for the super-detector (*p* < .152) groups. The detector group mean for identifying fear collected after 12 minutes was 5.67 correct and after 24 minutes was 4.39 correct. There were no significant differences for any group for happy (non-detectors, *p* < .108; detectors, *p* < .098; super-detectors, *p* < .729) but the trends result from slightly higher accuracy for the 12-minute collections. Our prediction that a longer collection time would increase accuracy is not supported.

A separate analysis was performed on the data from the exposure-to-the-label task. In this task either the happy or fear chemosignals were presented and the participant was asked which was collected early and which late. Participants were given feedback. It was possible that participants would learn which chemosignal was early or late and this would demonstrate that they were discriminable. A repeated measures ANOVA with gender as an independent variable, happy and fear trials as within variables, and blocks (3) of trials as the repeated variable produced no significant differences for gender (*F*(1) = .793, ns) nor for mood variable (*F*(1) = .466, ns) nor for blocks of trials (*F*(2) = .531, ns). This test indicates that participants did not learn to identify the 12 and 24 minute samples.

## Discussion

The results of this study show that chemosignals can be explicitly identified by many people as a signal for mood. It may not be a literary hyperbole that the detective hero entering a dark alley whispers, “Stay back. I smell fear, there is danger ahead.” A surprising number of people should be able to do this. This study successfully developed a method to examine individual differences in the identification of human mood using axillary chemosignals, called mood odors. We used statistical approaches adapted from behavioral genetics not used in this field previously. The model had unusually high reliability. The *r*_*tt*_ statistic first showed there was heterogeneity in the population. Secondly, GMM identified three distinct groups. The largest group (49.49%) was super-detectors, capable of consistent identification of both fear (anxiety) and happy (safety) chemosignals. The smallest group (17.98%) seemed anosmic for axillary odor under these conditions. They did not reliably identify either chemosignal or the sterile pad. The remaining group (32.52%) was slightly above chance levels in identifying moods. Longitudinal analyses (GMM) showed very high reliability for the placement of individuals within the three groups.

This is the first time that mood odor identification has been shown to be a reliable skill but it is not the first example of an unexpected body odor identification skill. Each of us has a unique odor signature that can be identified by ourselves more than 90% of the time [43] or by our near relatives [44] also with great reliability. The range of human olfactory skills is being expanded, as this study shows. The recent discoveries lead to many new questions, of course.

The focus of the present study is on explicit or “slow” and effortful processing of mood odor. It is not clear how it will impact our understanding of the rapidly growing research on implicit or “fast” processing [5] or on research pertaining to olfactics, that is, human olfactory communication [31]. That the brain responds without awareness (implicit processing) to odors was first clearly demonstrated by Pause [45, 46]. Her research has been the impetus for and has continued to support the position that awareness of body odor is not necessary for responses. It also shows that there are different brain processes for body odors than for common odors. The present study, in conjunction with prior research, has raised the question of whether there are separate roles for implicit and explicit processes in human mood odor communication or whether they interact or even whether the identification of mood odor is so effortful as to be seldom used.

The importance of dual processes, explicit and implicit, is apparent even outside olfaction and some of the concepts that apply in other areas may apply here as well. Kahneman [47] has reviewed extensive research on what he calls “slow” and “fast” processing from many disciplines. He notes that the interaction of explicit (akin to slow) and implicit (akin to fast) processes is complex and dynamic. Explicit responses may become implicit with practice as occurs with experts; that is, a response that has to be reasoned out initially or that requires effort may become automatic with practice. Neurological changes may even occur. Some implicit responses may be hard-wired and yet still show developmental changes and be affected by experience. Here, cultural expectations may be important. Ethnographers such as Low [48] show that in some cultures people are more aware of scent and even have terms for “scent identity.” Hall [49] noted that those of us in modern North America may be an extreme example of a scent deprived and scent ignorant culture. Knowledge of explicit mood odor, as well as identity body odor may be more difficult to detect in such cultures. In the area of olfactory process these hypotheses have hardly begun to be explored. One impediment has been the lack of methods to approach some aspects of explicit process and the consequent lack of information about individual differences. This study provides the groundwork for studying dual processing of mood odors.

There are a few studies that suggest explicit responses to body odor interact with other socioemotional behaviors. For example, Zhou and Chen [4] showed that “expert” explicit identification of roommate’s odor was related to socioemotional skills. Based on this study we might predict that “experts” or “super-detectors” for mood body odors have similar socioemotional expertise. On the other hand, it may be the case that explicit identification weakens some behaviors. Li, et al [50] found that odor only affected judgments of facial likeability when the odor was subliminal. When the odor was consciously detected, it had no effect. These studies leave open the question of whether a person who can identify a fear or happy mood odor is more likely to be sensitive to the contagious moods of others or is more likely to establish boundaries and be less affected.

### Secondary Analyses

The present study is one of the few to include semiochemicals collected from donors in happy mood inductions. There is a long history in studying negative emotions such as fear both in humans and other animals (for review [51]) and a smaller, but growing, literature on positive emotions (e.g., [52, 53]). Recent evidence has shown that both fear and happy mood odors are contagious [2, 3]. We had predicted that in explicit identification, fear would be more easily identified. The secondary analyses showed that the fear mood odors and the happy mood odors were equally distinguishable from the control. This suggested that we should reject the hypothesis; nevertheless, there is a possibility that the presentation of fear, happy, and control on each trial allowed for the participant to “know” either the fear (or the happy) as well as the control, leaving happy (or the fear) appearing to be “known” only by default. Different methods of presentation are needed to provide a more direct test before we conclude that each is separately easily identified. However, it is worth noting that both are distinguished from the non-body odor control by detectors, but not by non-detectors.

We had predicted that more women would be likely to identify the mood odors than men. As the task involved both a verbal report (labeling) and sensitivity to emotional differences, this seemed probable. However, there was only a trend for differences on this task. The task may well be one relying almost wholly on odor sensitivity and here there is less likelihood of differences. If the research were extended to varied social contexts, gender differences might be apparent [33]. Also, further studies with chemosignals collected from female donors may reveal some as yet unknown interactions.

We predicted that odors collected from donors after a longer period would be more easily identified than those collected for a shorter period. Within the boundaries of this study, chemosignals collected after 12 minutes were sufficient for identification and, generally, identification accuracy was the same for 12 and 24 minute periods. Exposure for a longer time period did not lead to more people being able to identify them. There was one instance in which the shorter period seemed more reliable. The detector group actually performed better with the 12-minute fear sample than the 24-minute sample. This result is congruent with research on implicit effects from fear mood odor [32]. As DeGroot and colleagues suggest, early collection may benefit from an adrenalin related process for the fear mood odors that produces more effective implicit responses. It is worth noting, however, that there was a trend for the happy mood odor early collection to also be easier to identify. It is possible that the longer video lost some effectiveness. It is also possible that participants became more effective in mood management over time and experienced other moods as a result.

The short exposure period presents some intriguing problems in gene regulation. Twelve minutes seems too short of a time for proteins (or modified proteins) to be made *de novo* and released from the skin cells. This implies that the proteins are “prepackaged” and only need to be “activated” or released (analogous to neurotransmitters). Studies at the cellular level may need to await isolation of active components of an emotional chemosignal response.

The issues with understanding threshold or intensity in the field of mood odors reminds us that further study is needed in understanding the production of the mood odors and their composition. It is possible that there is more than one combination of chemical product that can produce such effects, of course, and therefore there may be individual differences in the production of chemosensory signals as well as individual differences in the detection or identification of them. It is also possible that there are “suites” of chemicals used within groups of genetically-related individuals and that appropriate matches of related individuals would affect identification accuracy. It is clear that relatives can identify each other from body sweat and perhaps this extends to other communication [43].

### Methodological issues and future research

#### Long-term reliability of the phenotypic classification

It is possible that there is a genetic relationship supporting the phenotypically distinct detector groups, but it is also possible that the group differences are due to experience. The potential to train people who initially appear to be either inconsistent or unable to detect any mood odor still exists. The phenotypic model allows such individuals to be selected for training. The participants in this study were not given feedback on their choices of mood chemosignals so they were not likely to learn to identify the moods. We were only testing the existing skills in the population, not the potential for change. Providing motivation or a reward also may influence some individuals who appear to be non-detectors. It is also still important to test whether those who appear to be non-detectors in a labeling task are less responsive implicitly to mood odor. Though this initial study of individual differences provides significant new information, it leaves many questions.

In the next phase of research on individual differences in mood odor detection, it is important to determine if the individual differences shown in this study are stable over weeks or longer and how different contexts or chemosignals from different donors affect reliability. This is one more step in moving from an understanding of how phenotypic differences may reflect genetic or experiential differences in the population.

#### Establishing number of trials for reliability

There is an obvious advantage to using the r_tt_ statistic when heterogeneous population results are anticipated. This study demonstrates that repeated testing is needed to establish reliable individual differences for mood chemosignals. Whenever identification skills are meaningful in a research program, we recommend using the method shown here or another one that solves the same problem to establish reliability; we cannot assume that one or two trials are sufficient to identify individuals who may or may not be detectors. Even though in this study, about ten trials produced reliability, it also cannot be assumed that this is a standard. Each chemosensory stimulus may require its own analysis for homogeneity and trials needed for reliable identification of individuals.

Whereas multiple trials may be necessary to establish reliable phenotypes for mood odors, there may also be a disadvantage to using multiple trials in mood odor research. The mood odors are contagious [2, 3]. The longitudinal analysis in our study presented a four-group model that was almost as good as the three-group model that we discussed in more detail. The four group model indicated that a small part of the super-detector group deteriorated on the late trials. We may have induced moods by exposing participants to mood chemosignals and that induction may have affected the identification processes. This is a caution for future research and indicates a need for contextual controls.

#### GMM- Establishing phenotypic groups

To our knowledge this is the first use of longitudinal analysis in olfactory research. Hierarchical linear and empirical Bayesian models have emerged in several fields including genetics [15, 54, 55]. Such models classify heterogeneous data into discrete growth trajectory curves. It is useful to know the number of phenotypic groups in designing genetic research or to use groups to extend the information about an explicit reaction to the many implicit reactions that are already known for mood odors. There are also applications in environmental health assessments or applications for fragrance industries where people who may be super-detectors or highly sensitive may perform differently from non-detectors. The longitudinal modeling also obtained estimates for each individual showing the probability for belonging to each of the groups. In this case the degree of fit of individuals to the groups is very high. Almost all individuals were classified with at least a 98% probability of fit to one of the groups. Again, the statistical approaches provided by behavioral genetic modeling provide many advantages for olfactory research with surprisingly high reliabilities.

The methods from behavioral genetics that were used in this study may provide advantages for any testing of semiochemicals. They may be used to expand our understanding of dual processing of human odors. Broadly, testing may include consumer/industrial applications or mental health assessment, as well as a variety of medical issues of aging or physical illness. The results of the study, showing that there are phenotypically different groups in their ability to identify mood odors, may have far reaching effects.

## Supporting Information

S1 Text Sample Handling and Preparation for Individual Difference. (DOCX)

S2 Text Establishing reliable response patterns with the r_tt_. (DOCX)

S3 Text GMM Example. (DOCX)
