# Supplementary material for: Testing for Individual Differences in the Identification of Chemosignals for Fear and Happy: Phenotypic Super-Detectors, Detectors and Non-Detectors
Source: PLoS One. 2016 May 5;11(5):e0154495. doi: 10.1371/journal.pone.0154495 (PMC4858204; doi:10.1371/journal.pone.0154495)
Supplement: S1 Text — (DOCX) [file pone.0154495.s001.docx]

**S1. Text. Sample Handling and Preparation for Individual Difference**

All materials used for the preparation of sample presentation (e.g., presentation jars, scissors, storage jars) were prepared so as to minimize confounding odors (Wysocki, *per. comm*.) Materials were rinsed with reagent grade methylene-chloride, then rinsed with 95% methanol and finally oven dried. All samples and preparatory materials were handled with nylon lint free gloves. All preparatory materials were wrapped in heavy-grade aluminum foil cleaned as above and all work was performed on cleaned foil. Foil, scissors and gloves were always changed between types of samples to avoid any cross contamination.
