# Supplementary material for: Testing for Individual Differences in the Identification of Chemosignals for Fear and Happy: Phenotypic Super-Detectors, Detectors and Non-Detectors
Source: PLoS One. 2016 May 5;11(5):e0154495. doi: 10.1371/journal.pone.0154495 (PMC4858204; doi:10.1371/journal.pone.0154495)
Supplement: S2 Text — (DOCX) [file pone.0154495.s002.docx]

**S2. Text.** Establishing reliable response patterns with the r_tt_

According to Tryon (1957) one first conceptualizes some property, X, of a group of individuals (p 229). In this study, the property X will be the correct identification or sorting of fear and happy mood odors and a control odor on one trial. Next, one define[s] the property X_m_ in terms of objective specifications that directly lead to the taking of test-sample observations X_1_, X_2_, ... X_m_ believed to elicit the defined behavior, X” (p 229). In this study, such test samples (X_1_, X_2_…) are a series of trials in which participants correctly identify three (happy, fearful, and control) odors from the same donors. Lastly, one computes a “composite total score, X_t_“ (i.e., the number of correct choices over all trials). From such information we can calculate “within individual variance” and ultimately a reliability score r_tt_ (see Tyron 1957 for computational details) for the detection of fear and happy odors. Using this method, reliability is calculated for every trial and using this we can determine the exact number of trials needed to reach a specified reliability. A high ***r_tt_*** score means that observed phenotypic differences were not due to chance but demonstrate reliable patterns of response. The formula for r_tt_ is

$$r_{tt}=\frac{n}{n-1}\left( 1-\frac{\sum v_{i}}{v_{t}} \right)$$

For each trial (1 to n) we can compute a sample variance V_i_ (i = 1, 2, 3, 4, ....., n). In addition, for each composite score X**_t_** we can calculate a corresponding variance (V_t_) for the composite score. Where **n** is the number of trials and V**_t_** is the variance of the composite score in trial **n**. **V_i_** is the sum of the single trial variances (**V_1_ + V_2_ + ... V_n_**).
