# Supplementary material for: Testing for Individual Differences in the Identification of Chemosignals for Fear and Happy: Phenotypic Super-Detectors, Detectors and Non-Detectors
Source: PLoS One. 2016 May 5;11(5):e0154495. doi: 10.1371/journal.pone.0154495 (PMC4858204; doi:10.1371/journal.pone.0154495)
Supplement: S3 Text — (DOCX) [file pone.0154495.s003.docx]

**S3. Text. GMM Example**

To help clarify how we use GMM, we provide an example. Note that all the values in this example are all fictitious. This is an example provided by Derek Gordon, used with his permission:

Suppose we have four individuals who were given 4 trials per time period using the methods described in the paper. For simplicity, we’ll use the time-period values *t* = 0, 10, 20, 30, and 40 minutes, indicating when they performed the experiments. Since each experiment was scored as 0 or 1, the maximum number of correct trials at each time-period point is 4. When we apply the GMM method, we estimate that the optimal number of sub-groups is 2, with the corresponding curves where the random error follows a $N\left( 0,\varepsilon^{2} \right)$; that is, a normal distribution with mean 0 and error variance $\varepsilon^{2}=0.0012.$

$$\begin{matrix} {TG}_{1}\left( t \right)=1.25+0.068 t+N\left( 0,\varepsilon^{2} \right), \\ {TG}_{2}\left( t \right)=0.05+0.005 t+N\left( 0,\varepsilon^{2} \right), \end{matrix}$$

A graph of the two trajectory curves (with random error) may look like this:

It should be clear from studying this picture that individuals in sub-group ${TG}_{1}$ clearly seems to have a higher number of correct answers as time goes on. The individuals in sub-group ${TG}_{2}$ do not appear to substantially improve their scores over the time of the study.

Furthermore, if we create a chart of the individual BPPs, we might observe something like:

| **Individual** | ***TG*_1_ BPP** | ***TG*_2_ BPP** |
| --- | --- | --- |
| 1 | 0.99 | 0.01 |
| 2 | 0.02 | 0.98 |
| 3 | 0.45 | 0.55 |
| 4 | 0.15 | 0.85 |

From this chart, we observe that individual 1 is almost certainly in${TG}_{1}$, while individual 2 is almost certainly in ${TG}_{2}$. However, we cannot be sure to what sub-group individual 3 belongs, and while individual 4 has an 85% probability of belonging to ${TG}_{2}$, there is still a 15% probability that s/he belongs to ${TG}_{1}$. Thus, in some instances, classification may be imprecise. Finally, we obtain an estimate that 35% of the individuals belong to ${TG}_{1}$, while 65% belong to ${TG}_{2}$.
